# Bonvalotidine A acetone solvate from *Delphinium bonvalotii* Franch

**DOI:** 10.1107/S1600536810047562

**Published:** 2010-11-27

**Authors:** Shu-Hua Li, Feng-Zheng Chen

**Affiliations:** a Department of Chemistry and Life Sciences, Leshan Teachers College, Leshan 614004, People’s Republic of China

## Abstract

The title compound (systematic name: 5,6β-dihy­droxy-1α,14α,16β-trimeth­oxy-4-methyl-7β,8-methyl­enedi­oxy-20-ethyl­aconitan-6-yl acetate acetone monosolvate), C_27_H_41_NO_8_·C_3_H_6_O, was isolated from *Delphinium bonvalotii* Franch, and is a typical C_19_-diterpenoid alkaloid. The mol­ecule has a lycoctonine carbon skeleton with four six-membered rings and three five-membered rings. Three six-membered rings adopt the chair conformations while the fourth adopts a boat conformation, while the five-membered rings have the envelope conformations. The solvent mol­ecule links with the organic mol­ecule *via* a classical O—H⋯O hydrogen bond. Weak C—H⋯O hydrogen bonding is present in the structure. An intra­molecular O—H⋯O hydrogen bond also occurs.

## Related literature

For the chemical structure of the title compound established from NMR and MS data, see: He *et al.* (2006 ▶). For the crystal structures of related C_19_-diterpenoid alkaloids, see: Wang *et al.* (2009 ▶).
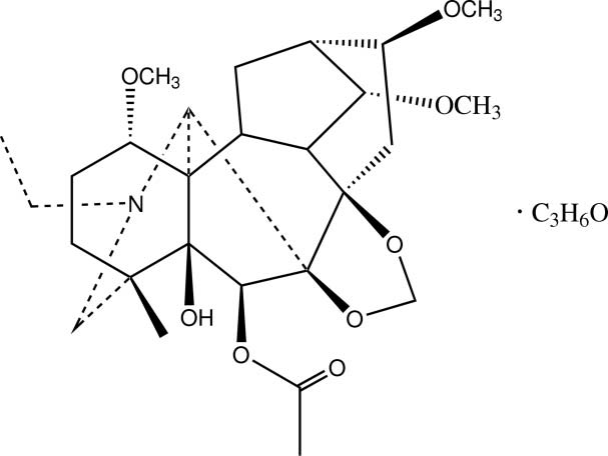


## Experimental

### 

#### Crystal data

C_27_H_41_NO_8_·C_3_H_6_O
*M* *_r_* = 565.69
Orthorhombic, 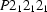

*a* = 8.4260 (8) Å
*b* = 9.5546 (7) Å
*c* = 35.237 (3) Å
*V* = 2836.8 (4) Å^3^
*Z* = 4
Mo *K*α radiation
μ = 0.10 mm^−1^
*T* = 120 K
0.42 × 0.36 × 0.21 mm

#### Data collection

Oxford Diffraction Xcalibur Eos diffractometer
9839 measured reflections
2930 independent reflections
2690 reflections with *I* > 2σ(*I*)
*R*_int_ = 0.022

#### Refinement

*R*[*F* ^2^ > 2σ(*F* ^2^)] = 0.044
*wR*(*F* ^2^) = 0.104
*S* = 1.08
2930 reflections
373 parameters
H atoms treated by a mixture of independent and constrained refinement
Δρ_max_ = 0.69 e Å^−3^
Δρ_min_ = −0.27 e Å^−3^

### 

Data collection: *CrysAlis CCD* (Oxford Diffraction, 2008 ▶); cell refinement: *CrysAlis RED* (Oxford Diffraction, 2008 ▶); data reduction: *CrysAlis RED*; program(s) used to solve structure: *SHELXS97* (Sheldrick, 2008 ▶); program(s) used to refine structure: *SHELXL97* (Sheldrick, 2008 ▶); molecular graphics: *OLEX2* (Dolomanov *et al.* 2009 ▶); software used to prepare material for publication: *SHELXL97*.

## Supplementary Material

Crystal structure: contains datablocks I, global. DOI: 10.1107/S1600536810047562/xu5072sup1.cif

Structure factors: contains datablocks I. DOI: 10.1107/S1600536810047562/xu5072Isup2.hkl

Additional supplementary materials:  crystallographic information; 3D view; checkCIF report

## Figures and Tables

**Table 1:** Hydrogen-bond geometry (Å, °)

*D*—H⋯*A*	*D*—H	H⋯*A*	*D*⋯*A*	*D*—H⋯*A*
O2—H2⋯O3	0.99 (5)	2.00 (5)	2.605 (3)	118 (4)
O2—H2⋯O9	0.99 (5)	2.13 (5)	2.936 (4)	138 (4)
C24—H24*A*⋯O9	0.98	2.53	3.427 (5)	153
C25—H25*A*⋯O2^i^	0.99	2.36	3.344 (4)	170

Symmetry code: (i) 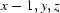.

**Fig. 1.**
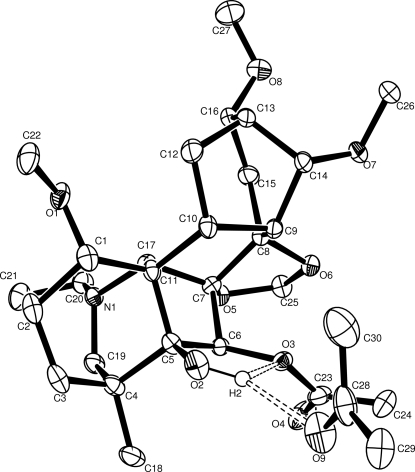
The molecular structure of thetitle compound with 30% probabiliy displacement ellipsoids for no-H atoms. Hatoms have been omitted clarity. Dashed line indicates hydrogen bonding.

## supplementary crystallographic information

### Comment

The title compound, bonvalotidine A, was previously isolated from *Delphinium bonvalotii* Franch (He *et al.* 2006), and its structure was established from the NMR and MS data. In our recent investigation, it was isolation from the root of *Delphinium bonvalotii* Franch collected in the E'mei Mountain, Sichuan Province of P. *R*. China in September, 2009. A nd its crystal structure was determined. The naming conforming referred to the literature (Wang *et al.* 2009). The molecular structure of the title compound is shown in Fig. 1. Six-membered rings A (C1/C2/C3/C4/C5/C11) and B (C7/C8/C9/C10/C11/C17) adopt chair conformations; six-membered ring D (C8/C9/C14/C13/C16/C15) adopt a boat conformation; six-membered N-containing heterocyclic ring E (C4/C5/C11/C17/N1/C19) displays the same chair conformation; five-membered rings C (C9/C10/C12/C13/C14) and F (C5/C6/C7/C17/C11) adopt an envelope conformation. While the five-membered O-containing heterocyclic G (O5/C7/C8/O6/C25) displays an envelope conformation.

The crystal structure contains intermolecular O—H···O hydrogen bond between the hydroxyl group and carbonyl O atom. The lattice acetone molecule links with the organic molecule *via* O—H···O hydrogen is present in the crystal structure (Table 1).

### Experimental

The title compound was isolated from the root of *Delphinium bonvalotii* Franch. Single crystals suitable for X-ray structure analysis were obtained by slow evaporation from an acetone solution at room temperature.

### Refinement

Hydroxy H atom was located in a difference Fourier map and refined isotropically. Other H atoms were located geometrically with C—H = 0.98–1.00 Å, and refined using a riding model with *U*_iso_(H) = 1.2 *U*_eq_(C). As no significant anomalous scatterings, Friedel pairs were merged.

### Crystal data

**Table:**

C_27_H_41_NO_8_·C_3_H_6_O	*F*(000) = 1224
*M**_r_* = 565.69	*D*_x_ = 1.324 Mg m^−^^3^
Orthorhombic, *P*2_1_2_1_2_1_	Mo *K*α radiation, λ = 0.71073 Å
Hall symbol: P 2ac 2ab	Cell parameters from 6568 reflections
*a* = 8.4260 (8) Å	θ = 3.1–25.0°
*b* = 9.5546 (7) Å	µ = 0.10 mm^−^^1^
*c* = 35.237 (3) Å	*T* = 120 K
*V* = 2836.8 (4) Å^3^	Block, colorless
*Z* = 4	0.42 × 0.36 × 0.21 mm

### Data collection

**Table:**

Oxford Diffraction Xcalibur Eos diffractometer	2690 reflections with *I* > 2σ(*I*)
Radiation source: fine-focus sealed tube	*R*_int_ = 0.022
graphite	θ_max_ = 25.2°, θ_min_ = 3.2°
ω scan	*h* = −10→9
9839 measured reflections	*k* = −11→11
2930 independent reflections	*l* = −22→42

### Refinement

**Table:**

Refinement on *F*^2^	Primary atom site location: structure-invariant direct methods
Least-squares matrix: full	Secondary atom site location: difference Fourier map
*R*[*F*^2^ > 2σ(*F*^2^)] = 0.044	Hydrogen site location: inferred from neighbouring sites
*wR*(*F*^2^) = 0.104	H atoms treated by a mixture of independent and constrained refinement
*S* = 1.08	*w* = 1/[σ^2^(*F*_o_^2^) + (0.0438*P*)^2^ + 2.2374*P*] where *P* = (*F*_o_^2^ + 2*F*_c_^2^)/3
2930 reflections	(Δ/σ)_max_ = 0.002
373 parameters	Δρ_max_ = 0.69 e Å^−^^3^
0 restraints	Δρ_min_ = −0.27 e Å^−^^3^

### Special details

**Table:**

Geometry. All e.s.d.'s (except the e.s.d. in the dihedral angle between two l.s. planes) are estimated using the full covariance matrix. The cell e.s.d.'s are taken into account individually in the estimation of e.s.d.'s in distances, angles and torsion angles; correlations between e.s.d.'s in cell parameters are only used when they are defined by crystal symmetry. An approximate (isotropic) treatment of cell e.s.d.'s is used for estimating e.s.d.'s involving l.s. planes.
Refinement. Refinement of *F*^2^ against ALL reflections. The weighted *R*-factor *wR* and goodness of fit *S* are based on *F*^2^, conventional *R*-factors *R* are based on *F*, with *F* set to zero for negative *F*^2^. The threshold expression of *F*^2^ > σ(*F*^2^) is used only for calculating *R*-factors(gt) *etc*. and is not relevant to the choice of reflections for refinement. *R*-factors based on *F*^2^ are statistically about twice as large as those based on *F*, and *R*- factors based on ALL data will be even larger.

### Fractional atomic coordinates and isotropic or equivalent isotropic displacement parameters (Å^2^)

**Table:**

	*x*	*y*	*z*	*U*_iso_*/*U*_eq_	
O1	0.6059 (3)	0.7225 (2)	0.47254 (6)	0.0246 (5)	
O2	0.7919 (3)	0.6104 (3)	0.35691 (7)	0.0269 (6)	
O3	0.5491 (3)	0.5711 (2)	0.31240 (6)	0.0184 (5)	
O4	0.4301 (4)	0.3889 (3)	0.28401 (7)	0.0398 (7)	
O5	0.2412 (3)	0.4687 (2)	0.36445 (6)	0.0189 (5)	
O6	0.2372 (3)	0.6543 (2)	0.32229 (6)	0.0186 (5)	
O7	0.2550 (3)	0.9818 (2)	0.32957 (6)	0.0223 (5)	
O8	0.0416 (3)	0.9541 (3)	0.40687 (7)	0.0329 (6)	
O9	0.8945 (3)	0.6682 (3)	0.27882 (8)	0.0436 (7)	
N1	0.4624 (3)	0.4363 (3)	0.43288 (7)	0.0173 (6)	
C1	0.6960 (4)	0.6670 (3)	0.44119 (9)	0.0231 (7)	
H1	0.7796	0.7371	0.4345	0.028*	
C2	0.7798 (4)	0.5360 (4)	0.45604 (10)	0.0271 (8)	
H2A	0.8683	0.5645	0.4728	0.033*	
H2B	0.7041	0.4806	0.4714	0.033*	
C3	0.8446 (4)	0.4447 (4)	0.42437 (10)	0.0271 (8)	
H3A	0.8870	0.3568	0.4353	0.033*	
H3B	0.9332	0.4940	0.4116	0.033*	
C4	0.7166 (4)	0.4097 (3)	0.39515 (9)	0.0214 (7)	
C5	0.6632 (4)	0.5480 (3)	0.37573 (9)	0.0182 (7)	
C6	0.5217 (4)	0.5115 (3)	0.34940 (8)	0.0161 (6)	
H6	0.5173	0.4074	0.3466	0.019*	
C7	0.3717 (4)	0.5591 (3)	0.37131 (9)	0.0151 (6)	
C8	0.3050 (4)	0.7005 (3)	0.35823 (9)	0.0180 (7)	
C9	0.4386 (4)	0.8063 (3)	0.35323 (8)	0.0161 (6)	
H9	0.4963	0.7901	0.3288	0.019*	
C10	0.5551 (4)	0.7971 (3)	0.38791 (9)	0.0191 (7)	
H10	0.6599	0.8331	0.3789	0.023*	
C11	0.5873 (4)	0.6482 (3)	0.40582 (9)	0.0169 (7)	
C12	0.4868 (5)	0.9106 (3)	0.41606 (9)	0.0287 (8)	
H12A	0.4622	0.8675	0.4409	0.034*	
H12B	0.5654	0.9862	0.4200	0.034*	
C13	0.3358 (4)	0.9694 (3)	0.39794 (9)	0.0255 (8)	
H13	0.3195	1.0693	0.4054	0.031*	
C14	0.3782 (4)	0.9577 (3)	0.35576 (9)	0.0204 (7)	
H14	0.4680	1.0229	0.3501	0.024*	
C15	0.1656 (4)	0.7551 (3)	0.38249 (10)	0.0232 (7)	
H15B	0.0782	0.7794	0.3649	0.028*	
H15A	0.1278	0.6763	0.3983	0.028*	
C16	0.1906 (4)	0.8800 (3)	0.40873 (9)	0.0257 (8)	
H16	0.2056	0.8445	0.4352	0.031*	
C17	0.4316 (4)	0.5675 (3)	0.41273 (8)	0.0158 (6)	
H17	0.3575	0.6267	0.4280	0.019*	
C18	0.7886 (4)	0.3079 (4)	0.36619 (10)	0.0284 (8)	
H18B	0.8907	0.3444	0.3573	0.043*	
H18C	0.7162	0.2978	0.3446	0.043*	
H18A	0.8047	0.2164	0.3782	0.043*	
C19	0.5701 (4)	0.3402 (3)	0.41337 (9)	0.0202 (7)	
H19B	0.5099	0.2908	0.3933	0.024*	
H19A	0.6069	0.2690	0.4318	0.024*	
C20	0.3177 (4)	0.3659 (3)	0.44604 (9)	0.0234 (7)	
H20B	0.2703	0.3131	0.4247	0.028*	
H20A	0.2397	0.4371	0.4543	0.028*	
C21	0.3508 (5)	0.2660 (4)	0.47871 (10)	0.0301 (8)	
H21C	0.4115	0.1856	0.4694	0.045*	
H21B	0.2502	0.2336	0.4895	0.045*	
H21A	0.4122	0.3146	0.4983	0.045*	
C22	0.6932 (5)	0.8197 (4)	0.49485 (10)	0.0325 (9)	
H22B	0.7969	0.7796	0.5012	0.049*	
H22A	0.6346	0.8399	0.5182	0.049*	
H22C	0.7084	0.9064	0.4805	0.049*	
C23	0.4946 (4)	0.5004 (3)	0.28192 (9)	0.0241 (7)	
C24	0.5246 (5)	0.5798 (4)	0.24627 (9)	0.0305 (9)	
H24C	0.5160	0.5165	0.2245	0.046*	
H24A	0.6313	0.6204	0.2471	0.046*	
H24B	0.4460	0.6549	0.2438	0.046*	
C25	0.1621 (4)	0.5249 (3)	0.33175 (9)	0.0215 (7)	
H25B	0.1700	0.4587	0.3102	0.026*	
H25A	0.0484	0.5406	0.3374	0.026*	
C26	0.2066 (5)	1.1253 (3)	0.32951 (10)	0.0296 (8)	
H26A	0.1479	1.1457	0.3529	0.044*	
H26C	0.1383	1.1429	0.3075	0.044*	
H26B	0.3006	1.1856	0.3281	0.044*	
C27	0.0114 (5)	1.0354 (4)	0.43882 (11)	0.0390 (9)	
H27A	0.0191	0.9770	0.4616	0.059*	
H27C	−0.0955	1.0752	0.4370	0.059*	
H27B	0.0895	1.1112	0.4403	0.059*	
C28	0.9445 (5)	0.7871 (4)	0.27569 (11)	0.0359 (9)	
C29	1.0840 (5)	0.8220 (5)	0.25177 (11)	0.0398 (10)	
H29C	1.1123	0.7409	0.2362	0.060*	
H29A	1.1739	0.8469	0.2681	0.060*	
H29B	1.0580	0.9013	0.2353	0.060*	
C30	0.8676 (6)	0.9039 (5)	0.29680 (14)	0.0504 (12)	
H30B	0.7676	0.8711	0.3079	0.076*	
H30C	0.8460	0.9814	0.2793	0.076*	
H30A	0.9384	0.9361	0.3171	0.076*	
H2	0.769 (5)	0.618 (5)	0.3296 (13)	0.059 (14)*	

### Atomic displacement parameters (Å^2^)

**Table:**

	*U*^11^	*U*^22^	*U*^33^	*U*^12^	*U*^13^	*U*^23^
O1	0.0318 (13)	0.0243 (12)	0.0178 (11)	−0.0064 (11)	−0.0067 (10)	0.0012 (10)
O2	0.0167 (12)	0.0326 (13)	0.0315 (14)	−0.0044 (10)	0.0015 (11)	0.0099 (11)
O3	0.0217 (11)	0.0168 (11)	0.0165 (10)	−0.0010 (9)	0.0041 (9)	−0.0009 (9)
O4	0.0657 (19)	0.0266 (13)	0.0271 (13)	−0.0164 (14)	0.0058 (14)	−0.0070 (11)
O5	0.0163 (10)	0.0202 (11)	0.0203 (11)	−0.0043 (10)	−0.0014 (9)	0.0020 (9)
O6	0.0179 (11)	0.0204 (11)	0.0174 (11)	−0.0032 (10)	−0.0009 (9)	0.0010 (9)
O7	0.0299 (12)	0.0156 (11)	0.0214 (11)	0.0038 (10)	−0.0032 (11)	0.0038 (9)
O8	0.0400 (14)	0.0306 (13)	0.0281 (13)	0.0117 (13)	0.0030 (12)	−0.0015 (12)
O9	0.0386 (16)	0.0432 (16)	0.0491 (17)	−0.0146 (14)	−0.0111 (14)	0.0109 (14)
N1	0.0213 (13)	0.0133 (12)	0.0175 (12)	−0.0007 (11)	0.0019 (11)	0.0027 (11)
C1	0.0236 (17)	0.0188 (16)	0.0270 (17)	−0.0058 (14)	−0.0051 (15)	0.0059 (14)
C2	0.0289 (18)	0.0255 (17)	0.0268 (17)	−0.0038 (16)	−0.0118 (16)	0.0082 (15)
C3	0.0211 (16)	0.0280 (17)	0.0323 (19)	0.0018 (16)	−0.0058 (16)	0.0121 (16)
C4	0.0194 (16)	0.0182 (15)	0.0265 (17)	0.0022 (14)	0.0038 (14)	0.0054 (14)
C5	0.0142 (15)	0.0175 (15)	0.0230 (16)	−0.0006 (13)	0.0002 (14)	0.0056 (14)
C6	0.0198 (15)	0.0131 (14)	0.0153 (15)	−0.0009 (13)	0.0045 (13)	0.0031 (12)
C7	0.0155 (15)	0.0146 (14)	0.0152 (15)	−0.0039 (13)	0.0002 (13)	−0.0016 (13)
C8	0.0195 (16)	0.0187 (15)	0.0157 (15)	0.0025 (13)	−0.0004 (13)	0.0001 (13)
C9	0.0215 (15)	0.0149 (14)	0.0118 (14)	0.0003 (13)	−0.0011 (13)	0.0013 (12)
C10	0.0260 (17)	0.0144 (15)	0.0171 (15)	−0.0048 (14)	−0.0060 (14)	0.0024 (13)
C11	0.0192 (16)	0.0148 (15)	0.0166 (15)	−0.0022 (13)	−0.0038 (13)	0.0036 (12)
C12	0.048 (2)	0.0183 (16)	0.0202 (16)	0.0011 (17)	−0.0103 (17)	−0.0036 (14)
C13	0.043 (2)	0.0161 (15)	0.0170 (16)	0.0085 (16)	−0.0071 (15)	−0.0017 (13)
C14	0.0310 (18)	0.0135 (14)	0.0167 (15)	0.0001 (14)	−0.0027 (15)	0.0020 (13)
C15	0.0216 (16)	0.0235 (16)	0.0246 (17)	0.0042 (15)	0.0049 (14)	0.0022 (14)
C16	0.037 (2)	0.0241 (17)	0.0161 (16)	0.0129 (16)	0.0013 (15)	0.0044 (14)
C17	0.0185 (15)	0.0127 (14)	0.0162 (15)	−0.0012 (13)	0.0005 (13)	−0.0014 (13)
C18	0.0256 (18)	0.0260 (17)	0.0334 (19)	0.0099 (15)	0.0078 (16)	0.0069 (15)
C19	0.0223 (16)	0.0166 (15)	0.0218 (16)	0.0007 (14)	0.0025 (14)	0.0053 (13)
C20	0.0256 (17)	0.0221 (16)	0.0227 (17)	−0.0020 (15)	0.0062 (15)	0.0038 (14)
C21	0.037 (2)	0.0283 (18)	0.0254 (19)	−0.0100 (17)	0.0003 (16)	0.0055 (15)
C22	0.041 (2)	0.0332 (19)	0.0238 (18)	−0.0082 (18)	−0.0118 (17)	0.0003 (16)
C23	0.0291 (17)	0.0205 (16)	0.0226 (17)	0.0002 (15)	0.0054 (15)	−0.0046 (14)
C24	0.036 (2)	0.036 (2)	0.0194 (16)	−0.0043 (18)	0.0030 (16)	0.0003 (15)
C25	0.0187 (15)	0.0217 (16)	0.0241 (17)	−0.0033 (14)	−0.0024 (14)	0.0023 (14)
C26	0.043 (2)	0.0215 (17)	0.0241 (18)	0.0090 (16)	−0.0003 (17)	0.0026 (14)
C27	0.036 (2)	0.044 (2)	0.037 (2)	0.007 (2)	0.0103 (18)	−0.0029 (18)
C28	0.0307 (19)	0.041 (2)	0.036 (2)	−0.0108 (18)	−0.0177 (18)	0.0152 (19)
C29	0.043 (2)	0.045 (2)	0.032 (2)	−0.008 (2)	−0.0068 (19)	0.0083 (19)
C30	0.041 (2)	0.048 (3)	0.062 (3)	−0.003 (2)	0.001 (2)	0.017 (2)

### Geometric parameters (Å, °)

**Table:**

O1—C22	1.421 (4)	C12—C13	1.531 (5)
O1—C1	1.442 (4)	C12—H12A	0.9900
O2—C5	1.404 (4)	C12—H12B	0.9900
O2—H2	0.99 (5)	C13—C14	1.533 (4)
O3—C23	1.349 (4)	C13—C16	1.540 (5)
O3—C6	1.441 (3)	C13—H13	1.0000
O4—C23	1.198 (4)	C14—H14	1.0000
O5—C7	1.419 (4)	C15—C16	1.524 (5)
O5—C25	1.436 (4)	C15—H15B	0.9900
O6—C25	1.429 (4)	C15—H15A	0.9900
O6—C8	1.457 (4)	C16—H16	1.0000
O7—C14	1.408 (4)	C17—H17	1.0000
O7—C26	1.431 (4)	C18—H18B	0.9800
O8—C27	1.391 (4)	C18—H18C	0.9800
O8—C16	1.443 (4)	C18—H18A	0.9800
O9—C28	1.217 (5)	C19—H19B	0.9900
N1—C19	1.462 (4)	C19—H19A	0.9900
N1—C17	1.464 (4)	C20—C21	1.521 (4)
N1—C20	1.468 (4)	C20—H20B	0.9900
C1—C2	1.530 (5)	C20—H20A	0.9900
C1—C11	1.557 (4)	C21—H21C	0.9800
C1—H1	1.0000	C21—H21B	0.9800
C2—C3	1.518 (5)	C21—H21A	0.9800
C2—H2A	0.9900	C22—H22B	0.9800
C2—H2B	0.9900	C22—H22A	0.9800
C3—C4	1.528 (5)	C22—H22C	0.9800
C3—H3A	0.9900	C23—C24	1.489 (5)
C3—H3B	0.9900	C24—H24C	0.9800
C4—C18	1.535 (5)	C24—H24A	0.9800
C4—C19	1.542 (4)	C24—H24B	0.9800
C4—C5	1.555 (4)	C25—H25B	0.9900
C5—C6	1.550 (4)	C25—H25A	0.9900
C5—C11	1.566 (4)	C26—H26A	0.9800
C6—C7	1.549 (4)	C26—H26C	0.9800
C6—H6	1.0000	C26—H26B	0.9800
C7—C8	1.534 (4)	C27—H27A	0.9800
C7—C17	1.547 (4)	C27—H27C	0.9800
C8—C9	1.523 (4)	C27—H27B	0.9800
C8—C15	1.544 (4)	C28—C29	1.484 (6)
C9—C14	1.536 (4)	C28—C30	1.490 (6)
C9—C10	1.570 (4)	C29—H29C	0.9800
C9—H9	1.0000	C29—H29A	0.9800
C10—C12	1.578 (4)	C29—H29B	0.9800
C10—C11	1.579 (4)	C30—H30B	0.9800
C10—H10	1.0000	C30—H30C	0.9800
C11—C17	1.541 (4)	C30—H30A	0.9800
			
C22—O1—C1	113.1 (3)	C13—C14—H14	109.0
C5—O2—H2	110 (3)	C9—C14—H14	109.0
C23—O3—C6	117.9 (2)	C16—C15—C8	119.7 (3)
C7—O5—C25	105.6 (2)	C16—C15—H15B	107.4
C25—O6—C8	103.5 (2)	C8—C15—H15B	107.4
C14—O7—C26	111.6 (3)	C16—C15—H15A	107.4
C27—O8—C16	113.3 (3)	C8—C15—H15A	107.4
C19—N1—C17	114.8 (2)	H15B—C15—H15A	106.9
C19—N1—C20	112.1 (2)	O8—C16—C15	103.7 (3)
C17—N1—C20	113.5 (2)	O8—C16—C13	114.1 (3)
O1—C1—C2	106.4 (3)	C15—C16—C13	113.2 (3)
O1—C1—C11	110.2 (3)	O8—C16—H16	108.5
C2—C1—C11	116.8 (3)	C15—C16—H16	108.5
O1—C1—H1	107.7	C13—C16—H16	108.5
C2—C1—H1	107.7	N1—C17—C11	110.7 (2)
C11—C1—H1	107.7	N1—C17—C7	118.1 (2)
C3—C2—C1	112.6 (3)	C11—C17—C7	98.9 (2)
C3—C2—H2A	109.1	N1—C17—H17	109.5
C1—C2—H2A	109.1	C11—C17—H17	109.5
C3—C2—H2B	109.1	C7—C17—H17	109.5
C1—C2—H2B	109.1	C4—C18—H18B	109.5
H2A—C2—H2B	107.8	C4—C18—H18C	109.5
C2—C3—C4	111.5 (3)	H18B—C18—H18C	109.5
C2—C3—H3A	109.3	C4—C18—H18A	109.5
C4—C3—H3A	109.3	H18B—C18—H18A	109.5
C2—C3—H3B	109.3	H18C—C18—H18A	109.5
C4—C3—H3B	109.3	N1—C19—C4	115.0 (3)
H3A—C3—H3B	108.0	N1—C19—H19B	108.5
C3—C4—C18	107.9 (3)	C4—C19—H19B	108.5
C3—C4—C19	112.3 (3)	N1—C19—H19A	108.5
C18—C4—C19	108.7 (3)	C4—C19—H19A	108.5
C3—C4—C5	108.4 (3)	H19B—C19—H19A	107.5
C18—C4—C5	111.1 (3)	N1—C20—C21	112.0 (3)
C19—C4—C5	108.5 (2)	N1—C20—H20B	109.2
O2—C5—C6	114.0 (3)	C21—C20—H20B	109.2
O2—C5—C4	110.2 (2)	N1—C20—H20A	109.2
C6—C5—C4	107.2 (2)	C21—C20—H20A	109.2
O2—C5—C11	112.0 (3)	H20B—C20—H20A	107.9
C6—C5—C11	103.2 (2)	C20—C21—H21C	109.5
C4—C5—C11	109.9 (3)	C20—C21—H21B	109.5
O3—C6—C7	117.8 (2)	H21C—C21—H21B	109.5
O3—C6—C5	109.2 (2)	C20—C21—H21A	109.5
C7—C6—C5	105.3 (2)	H21C—C21—H21A	109.5
O3—C6—H6	108.1	H21B—C21—H21A	109.5
C7—C6—H6	108.1	O1—C22—H22B	109.5
C5—C6—H6	108.1	O1—C22—H22A	109.5
O5—C7—C8	101.6 (2)	H22B—C22—H22A	109.5
O5—C7—C17	116.4 (2)	O1—C22—H22C	109.5
C8—C7—C17	110.9 (2)	H22B—C22—H22C	109.5
O5—C7—C6	111.6 (2)	H22A—C22—H22C	109.5
C8—C7—C6	114.1 (2)	O4—C23—O3	123.4 (3)
C17—C7—C6	102.7 (2)	O4—C23—C24	125.6 (3)
O6—C8—C9	112.9 (2)	O3—C23—C24	111.0 (3)
O6—C8—C7	98.0 (2)	C23—C24—H24C	109.5
C9—C8—C7	110.4 (2)	C23—C24—H24A	109.5
O6—C8—C15	106.6 (2)	H24C—C24—H24A	109.5
C9—C8—C15	113.7 (3)	C23—C24—H24B	109.5
C7—C8—C15	114.2 (3)	H24C—C24—H24B	109.5
C8—C9—C14	112.0 (3)	H24A—C24—H24B	109.5
C8—C9—C10	109.6 (2)	O6—C25—O5	107.8 (2)
C14—C9—C10	102.4 (2)	O6—C25—H25B	110.2
C8—C9—H9	110.9	O5—C25—H25B	110.2
C14—C9—H9	110.9	O6—C25—H25A	110.2
C10—C9—H9	110.9	O5—C25—H25A	110.2
C9—C10—C12	102.9 (2)	H25B—C25—H25A	108.5
C9—C10—C11	118.0 (2)	O7—C26—H26A	109.5
C12—C10—C11	115.5 (2)	O7—C26—H26C	109.5
C9—C10—H10	106.6	H26A—C26—H26C	109.5
C12—C10—H10	106.6	O7—C26—H26B	109.5
C11—C10—H10	106.6	H26A—C26—H26B	109.5
C17—C11—C1	115.6 (2)	H26C—C26—H26B	109.5
C17—C11—C5	98.5 (2)	O8—C27—H27A	109.5
C1—C11—C5	111.9 (3)	O8—C27—H27C	109.5
C17—C11—C10	111.6 (2)	H27A—C27—H27C	109.5
C1—C11—C10	108.5 (2)	O8—C27—H27B	109.5
C5—C11—C10	110.5 (2)	H27A—C27—H27B	109.5
C13—C12—C10	107.1 (3)	H27C—C27—H27B	109.5
C13—C12—H12A	110.3	O9—C28—C29	122.4 (4)
C10—C12—H12A	110.3	O9—C28—C30	120.2 (4)
C13—C12—H12B	110.3	C29—C28—C30	117.4 (3)
C10—C12—H12B	110.3	C28—C29—H29C	109.5
H12A—C12—H12B	108.6	C28—C29—H29A	109.5
C12—C13—C14	100.6 (3)	H29C—C29—H29A	109.5
C12—C13—C16	110.7 (3)	C28—C29—H29B	109.5
C14—C13—C16	112.6 (3)	H29C—C29—H29B	109.5
C12—C13—H13	110.8	H29A—C29—H29B	109.5
C14—C13—H13	110.8	C28—C30—H30B	109.5
C16—C13—H13	110.8	C28—C30—H30C	109.5
O7—C14—C13	116.8 (3)	H30B—C30—H30C	109.5
O7—C14—C9	111.1 (2)	C28—C30—H30A	109.5
C13—C14—C9	101.7 (2)	H30B—C30—H30A	109.5
O7—C14—H14	109.0	H30C—C30—H30A	109.5
			
C22—O1—C1—C2	89.7 (3)	O2—C5—C11—C1	−72.0 (3)
C22—O1—C1—C11	−142.7 (3)	C6—C5—C11—C1	164.8 (2)
O1—C1—C2—C3	165.1 (3)	C4—C5—C11—C1	50.8 (3)
C11—C1—C2—C3	41.5 (4)	O2—C5—C11—C10	49.0 (3)
C1—C2—C3—C4	−52.8 (4)	C6—C5—C11—C10	−74.2 (3)
C2—C3—C4—C18	−175.4 (3)	C4—C5—C11—C10	171.8 (2)
C2—C3—C4—C19	−55.6 (4)	C9—C10—C11—C17	−47.6 (4)
C2—C3—C4—C5	64.2 (3)	C12—C10—C11—C17	74.5 (3)
C3—C4—C5—O2	61.2 (3)	C9—C10—C11—C1	−176.1 (3)
C18—C4—C5—O2	−57.2 (4)	C12—C10—C11—C1	−53.9 (4)
C19—C4—C5—O2	−176.6 (3)	C9—C10—C11—C5	60.9 (4)
C3—C4—C5—C6	−174.2 (2)	C12—C10—C11—C5	−176.9 (3)
C18—C4—C5—C6	67.4 (3)	C9—C10—C12—C13	3.4 (3)
C19—C4—C5—C6	−52.1 (3)	C11—C10—C12—C13	−126.5 (3)
C3—C4—C5—C11	−62.7 (3)	C10—C12—C13—C14	−31.9 (3)
C18—C4—C5—C11	178.8 (3)	C10—C12—C13—C16	87.4 (3)
C19—C4—C5—C11	59.4 (3)	C26—O7—C14—C13	68.7 (4)
C23—O3—C6—C7	−93.5 (3)	C26—O7—C14—C9	−175.3 (3)
C23—O3—C6—C5	146.6 (3)	C12—C13—C14—O7	169.9 (3)
O2—C5—C6—O3	−8.6 (3)	C16—C13—C14—O7	52.0 (4)
C4—C5—C6—O3	−130.9 (3)	C12—C13—C14—C9	48.8 (3)
C11—C5—C6—O3	113.1 (3)	C16—C13—C14—C9	−69.1 (3)
O2—C5—C6—C7	−136.0 (3)	C8—C9—C14—O7	−55.1 (3)
C4—C5—C6—C7	101.8 (3)	C10—C9—C14—O7	−172.4 (2)
C11—C5—C6—C7	−14.2 (3)	C8—C9—C14—C13	69.9 (3)
C25—O5—C7—C8	32.6 (3)	C10—C9—C14—C13	−47.3 (3)
C25—O5—C7—C17	153.2 (3)	O6—C8—C15—C16	−144.4 (3)
C25—O5—C7—C6	−89.4 (3)	C9—C8—C15—C16	−19.3 (4)
O3—C6—C7—O5	92.7 (3)	C7—C8—C15—C16	108.6 (3)
C5—C6—C7—O5	−145.3 (2)	C27—O8—C16—C15	155.1 (3)
O3—C6—C7—C8	−21.7 (4)	C27—O8—C16—C13	−81.3 (4)
C5—C6—C7—C8	100.2 (3)	C8—C15—C16—O8	144.4 (3)
O3—C6—C7—C17	−141.8 (3)	C8—C15—C16—C13	20.2 (4)
C5—C6—C7—C17	−19.9 (3)	C12—C13—C16—O8	155.7 (3)
C25—O6—C8—C9	159.4 (2)	C14—C13—C16—O8	−92.6 (3)
C25—O6—C8—C7	43.2 (3)	C12—C13—C16—C15	−86.1 (3)
C25—O6—C8—C15	−75.1 (3)	C14—C13—C16—C15	25.7 (4)
O5—C7—C8—O6	−46.9 (3)	C19—N1—C17—C11	−59.0 (3)
C17—C7—C8—O6	−171.3 (2)	C20—N1—C17—C11	170.3 (3)
C6—C7—C8—O6	73.4 (3)	C19—N1—C17—C7	53.9 (4)
O5—C7—C8—C9	−165.0 (2)	C20—N1—C17—C7	−76.8 (3)
C17—C7—C8—C9	70.6 (3)	C1—C11—C17—N1	−49.6 (3)
C6—C7—C8—C9	−44.8 (3)	C5—C11—C17—N1	69.7 (3)
O5—C7—C8—C15	65.4 (3)	C10—C11—C17—N1	−174.2 (2)
C17—C7—C8—C15	−59.0 (3)	C1—C11—C17—C7	−174.3 (3)
C6—C7—C8—C15	−174.3 (3)	C5—C11—C17—C7	−55.0 (3)
O6—C8—C9—C14	94.0 (3)	C10—C11—C17—C7	61.2 (3)
C7—C8—C9—C14	−157.4 (3)	O5—C7—C17—N1	49.7 (4)
C15—C8—C9—C14	−27.6 (4)	C8—C7—C17—N1	165.2 (3)
O6—C8—C9—C10	−153.1 (2)	C6—C7—C17—N1	−72.6 (3)
C7—C8—C9—C10	−44.6 (3)	O5—C7—C17—C11	169.0 (2)
C15—C8—C9—C10	85.3 (3)	C8—C7—C17—C11	−75.5 (3)
C8—C9—C10—C12	−92.5 (3)	C6—C7—C17—C11	46.8 (3)
C14—C9—C10—C12	26.5 (3)	C17—N1—C19—C4	42.6 (4)
C8—C9—C10—C11	35.9 (4)	C20—N1—C19—C4	174.0 (3)
C14—C9—C10—C11	154.9 (3)	C3—C4—C19—N1	77.9 (3)
O1—C1—C11—C17	−50.8 (3)	C18—C4—C19—N1	−162.8 (3)
C2—C1—C11—C17	70.8 (4)	C5—C4—C19—N1	−41.9 (4)
O1—C1—C11—C5	−162.5 (2)	C19—N1—C20—C21	70.1 (3)
C2—C1—C11—C5	−40.9 (4)	C17—N1—C20—C21	−157.8 (3)
O1—C1—C11—C10	75.3 (3)	C6—O3—C23—O4	−2.8 (5)
C2—C1—C11—C10	−163.1 (3)	C6—O3—C23—C24	176.8 (3)
O2—C5—C11—C17	165.9 (2)	C8—O6—C25—O5	−25.3 (3)
C6—C5—C11—C17	42.8 (3)	C7—O5—C25—O6	−5.7 (3)
C4—C5—C11—C17	−71.3 (3)		

### Hydrogen-bond geometry (Å, °)

**Table:**

*D*—H···*A*	*D*—H	H···*A*	*D*···*A*	*D*—H···*A*
O2—H2···O3	0.99 (5)	2.00 (5)	2.605 (3)	118 (4)
O2—H2···O9	0.99 (5)	2.13 (5)	2.936 (4)	138 (4)
C24—H24A···O9	0.98	2.53	3.427 (5)	153
C25—H25A···O2^i^	0.99	2.36	3.344 (4)	170

Symmetry codes: (i) *x*−1, *y*, *z*.
